# Supplementary material for: The evolution of whole-brain turbulent dynamics during recovery from traumatic brain injury
Source: Netw Neurosci. 2024 Apr 1;8(1):158–77. doi: 10.1162/netn_a_00346 (PMC10898780; doi:10.1162/netn_a_00346)
Supplement: Supplementary file 1 [file netn-8-1-158-s001.pdf]

Supporting Information

Outlier identification and denoising in the CONN toolbox

Outlier scans were identified as those that exceeded 3 standard deviations in z-scores from the global BOLD signal change (GSC) or with framewise displacement (FD) that exceeded 2 mm. The GSC timeseries was computed at each scan as the absolute value of the scan-to-scan change in global BOLD signal using SPM global BOLD signal definition. The GSC timeseries were then scaled to standard units within each run by subtracting their median value and dividing by 0.74 times their interquartile range (Whitfield-Gabrieli et al., 2011). The FD timeseries was defined as the maximum change in the position of six points placed at the centers of each face in a 140 x 180 x 115 mm bounding box around the brain and undergoing the same rotations and translations as the participant's head.

For denoising the timeseries, we used the default pipeline in the CONN toolbox (Nieto-Castanon, 2020) in order to characterize (noise components extraction) and minimize the effect of non-neural noise on the BOLD timeseries. Participant-specific minimally eroded white matter (WM) and cerebrospinal fluid (CSF) masks were generated through a one-voxel binary erosion of tissue masks obtained after segmentation. Next, noise components were extracted from these WM and CSF masks using principal components analysis following the anatomical aCompCor methods (Behzadi et al., 2007). Principal component from WM and CSF were computed after discounting motion and outlier effects. Ordinary least square regression removed from each voxel BOLD timeseries the effect of all identified noise components including 5 components from WM, 5 components from CSF, 12 estimated motion parameters (6 realignment parameters and their first order time derivatives), outlier scans, effect of session and its first level derivative convolved with the canonical hemodynamic response function, and constant and linear session effects (i.e., linear detrending).

Quality Control in the CONN toolbox

Participant-level visual quality control (QC) was done to evaluate possible patterns or other features that may be visible in the BOLD signal timeseries after denoising. We reviewed run-specific plots rendering carpetplots (Power, 2017) of fully preprocessed BOLD timeseries before and after denoising, together with the traces of GSC, FD and outliers timeseries. This inspection aimed to confirm that sudden and synchronized variations in signal intensity had been flagged as outliers, and that there were no visible residual large-scale patterns in the BOLD signal timeseries, which could indicate the persistence of global or widespread noise sources.
In addition, we computed functional connectivity values (Pearson's correlation coefficients) among all pairs from a fixed set of 1,000 random voxels within the MNI-space gray matter template mask in order to evaluate a relatively dense sample of connections from the whole-brain connectome. From these connections, we computed and displayed the distribution of functional connectivity (FC) values separately for each participant's functional run. Visual inspection of these distributions allows us to evaluate the relative presence of residual noise sources in the BOLD timeseries, which tend to shift the entire FC distribution towards positive values (false positives), altering the FC distribution center and overall shape in a manner that is highly variable across runs. In comparison, the relative absence of noise sources is expressed as FC distributions that appear relatively centered (with a small positive distribution mean and a distribution mode approximately at zero) and similar across different runs and participants (Nieto-Castanon 2020). After evaluating the distribution of functional connectivity after denoising, the number of noise components extracted from the WM was increased to 10.

Table S1. Turbulent and perturbational measures in Healthy Controls. Results from the repeated measures ANOVA with time as within-group factor and paired T-test (top table) and Wilcoxon signed rank test (bottom table). Schaefer parcellation 1000 nodes 7 RSNs.

**Measure**	**Effect**	**Statistic**	**pval**
Global Amplitude	Time x Lambda	$F_{(9,99)}$=1.471	0.169
Information Cascade Flow	Time x Lambda	$F_{(8,88)}$=1.131	0.351
Information Cascade	Time	$t_{(11)}$=-1.034	0.323
Information Transfer	Time x Lambda	$F_{(9,99)}$=0.349	0.946

**Measure**	**Z**	**pval**
Susceptibility	8.64	<0.0001
Information encoding capability	8.52	<0.0001

Table S2. Global Amplitude Turbulence in Healthy Controls (mean across session 1 and 2) vs TBI patients (3-,6- and 12-months). Results from the 4 (Group) x 10 (Lambda) ANOVA (top table) and post hoc tests (bottom table). Schaefer parcellation 1000 nodes 7 RSNs.

**Effect**	**$F_{(27,440)}$**	**p-val**	**Effect size ($\eta^2_{partial}$)**
**Group x Lambda**	2.534	<0.0001	0.135

**Lambda, Group**	**Lambda, Group**	**Mean Difference**	**t**	**pval**
**0.01, Healthy Controls**	**0.01, TBI at 3-months post-injury**	0.014	4.201	**0.018**
	**0.01, TBI at 6-months post-injury**	**0.024**	7.296	**<0.0001**
	**0.01, TBI at 12-months post-injury**	0.014	4.208	**0.017**
**0.03, Healthy Controls**	**0.03, TBI at 3-months post-injury**	0.015	4.760	**0.002**
	**0.03, TBI at 6-months post-injury**	**0.023**	7.108	**<0.0001**
	**0.03, TBI at 12-months post-injury**	0.014	4.152	**0.022**
**0.06, Healthy Controls**	**0.06, TBI at 3-months post-injury**	0.014	4.166	**0.020**
	**0.06, TBI at 6-months post-injury**	**0.017**	5.326	**<0.0001**
	0.06, TBI at 12-months post-injury	0.009	2.898	0.607
0.09, Healthy Controls	0.09, TBI at 3-months post-injury	0.010	3.166	0.386
	0.09, TBI at 6-months post-injury	0.012	3.676	0.108
	0.09, TBI at 12-months post-injury	0.006	1.925	0.998
0.12, Healthy Controls	0.12, TBI at 3-months post-injury	0.007	2.271	0.964
	0.12, TBI at 6-months post-injury	0.008	2.467	0.901
	0.12, TBI at 12-months post-injury	0.004	1.297	1.000
0.15, Healthy Controls	0.15, TBI at 3-months post-injury	0.005	1.592	1.000
	0.15, TBI at 6-months post-injury	0.005	1.641	1.000
	0.15, TBI at 12-months post-injury	0.003	1.015	1.000
0.18, Healthy Controls	0.18, TBI at 3-months post-injury	0.004	1.151	1.000
	0.18, TBI at 6-months post-injury	0.004	1.107	1.000
	0.18, TBI at 12-months post-injury	0.003	0.888	1.000
0.21, Healthy Controls	0.21, TBI at 3-months post-injury	0.003	0.871	1.000

	0.21, TBI at 6-months post-injury	0.002	0.754	1.000
	0.21, TBI at 12-months post-injury	0.003	0.793	1.000
0.24, Healthy Controls	0.24, TBI at 3-months post-injury	0.002	0.671	1.000
	0.24, TBI at 6-months post-injury	0.002	0.503	1.000
	0.24, TBI at 12-months post-injury	0.002	0.696	1.000
0.27, Healthy Controls	0.27, TBI at 3-months post-injury	0.002	0.521	1.000
	0.27, TBI at 6-months post-injury	0.001	0.324	1.000
	0.27, TBI at 12-months post-injury	0.002	0.602	1.000

Table S3. Information Cascade Flow in Healthy Controls (mean across session 1 and 2) vs TBI patients (3-,6- and 12-months). Results from the 4 (Group) x 9 (Lambda) ANOVA (top table) and post hoc tests (bottom table). Schaefer parcellation 1000 nodes 7 RSNs.

**Effect**	$F_{(24,396)}$	**p-val**	**Effect size ($\eta^2_{partial}$)**
Group x Lambda	3.007	<0.0001	0.154

**Lambda, Group**	**Lambda, Group**	**Mean Difference**	**t**	**pval**
**0.01, Healthy Controls**	**0.01, TBI at 3-months post-injury**	0.055	4.512	**0.004**
	**0.01, TBI at 6-months post-injury**	0.106	8.718	**0.000**
	**0.01, TBI at 12-months post-injury**	0.047	3.873	**0.049**
**0.03, Healthy Controls**	0.03, TBI at 3-months post-injury	0.038	3.098	0.394
	**0.03, TBI at 6-months post-injury**	0.064	5.303	**<0.0001**
	0.03, TBI at 12-months post-injury	0.033	2.700	0.716
0.06, Healthy Controls	0.06, TBI at 3-months post-injury	0.020	1.619	1.000
	0.06, TBI at 6-months post-injury	0.029	2.356	0.918
	0.06, TBI at 12-months post-injury	0.016	1.342	1.000
0.09, Healthy Controls	0.09, TBI at 3-months post-injury	0.013	1.040	1.000
	0.09, TBI at 6-months post-injury	0.015	1.273	1.000
	0.09, TBI at 12-months post-injury	0.010	0.812	1.000
0.12, Healthy Controls	0.12, TBI at 3-months post-injury	0.008	0.695	1.000
	0.12, TBI at 6-months post-injury	0.009	0.751	1.000
	0.12, TBI at 12-months post-injury	0.006	0.473	1.000
0.15, Healthy Controls	0.15, TBI at 3-months post-injury	0.006	0.461	1.000
	0.15, TBI at 6-months post-injury	0.006	0.479	1.000
	0.15, TBI at 12-months post-injury	0.003	0.266	1.000
0.18, Healthy Controls	0.18, TBI at 3-months post-injury	0.004	0.312	1.000
	0.18, TBI at 6-months post-injury	0.004	0.322	1.000
	0.18, TBI at 12-months post-injury	0.002	0.138	1.000
0.21, Healthy Controls	0.21, TBI at 3-months post-injury	0.003	0.219	1.000
	0.21, TBI at 6-months post-injury	0.003	0.229	1.000
	0.21, TBI at 12-months post-injury	7.924×10^{-4}	0.065	1.000
0.24, Healthy Controls	0.24, TBI at 3-months post-injury	0.002	0.167	1.000
	0.24, TBI at 6-months post-injury	0.002	0.169	1.000

	0.24, TBI at 12-months post-injury	3.200×10^{-4}	0.026	1.000

Table S4. Information Cascade in Healthy Controls (mean across session 1 and 2) vs TBI patients (3-,6- and 12-months). Results from the 4 one-way ANOVA (top table) and post hoc tests (bottom table). Schaefer parcellation 1000 nodes 7 RSNs.

Effect	$F_{(3,44)}$	p-val	Effect size ($\eta^2_{partial}$)
Group	7.061	<0.0001	0.325

Group 1	Group 2	Mean Difference	t	pval
**Healthy Controls**	**TBI at 3-months post-injury**	0.017	2.833	**0.034**
	**TBI at 6-months post-injury**	0.027	4.552	**<0.0001**
	TBI at 12-months post-injury	0.013	2.276	0.119
TBI at 3-months post-injury	TBI at 6-months post-injury	0.010	1.718	0.327
	TBI at 12-months post-injury	-0.003	-0.557	0.944
TBI at 6-months post-injury	TBI at 12-months post-injury	-0.013	-2.275	0.119

Table S5. Information Transfer in Healthy Controls Healthy Controls (mean across session 1 and 2) vs TBI patients (3-,6- and 12-months). Results from the 4 (Group) x 10 (Lambda) ANOVA (top table) and post hoc tests (bottom table). Schaefer parcellation 1000 nodes 7 RSNs.

Effect	$F_{(27,440)}$	p-val	Effect size ($\eta^2_{partial}$)
Group x Lambda	2.325	<0.0001	0.125

Lambda, Group	Lambda, Group	Mean Difference	t	pval
0.01, Healthy Controls	0.01, TBI at 3-months post-injury	-4.843×10^{-4}	-0.023	1.000
	0.01, TBI at 6-months post-injury	-0.001	-0.057	1.000
	0.01, TBI at 12-months post-injury	-6.715×10^{-4}	-0.032	1.000
0.03, Healthy Controls	0.03, TBI at 3-months post-injury	-0.007	-0.321	1.000
	0.03, TBI at 6-months post-injury	-0.011	-0.514	1.000
	0.03, TBI at 12-months post-injury	-0.007	-0.324	1.000
0.06, Healthy Controls	0.06, TBI at 3-months post-injury	-0.026	-1.225	1.000
	0.06, TBI at 6-months post-injury	-0.028	-1.321	1.000
	0.06, TBI at 12-months post-injury	-0.020	-0.965	1.000
0.09, Healthy Controls	0.09, TBI at 3-months post-injury	-0.048	-2.313	0.954
	0.09, TBI at 6-months post-injury	-0.042	-2.014	0.994
	0.09, TBI at 12-months post-injury	-0.033	-1.605	1.000
0.12, Healthy Controls	0.12, TBI at 3-months post-injury	-0.073	-3.500	0.177
	0.12, TBI at 6-months post-injury	-0.055	-2.627	0.813
	0.12, TBI at 12-months post-injury	-0.044	-2.110	0.988
**0.15, Healthy Controls**	**0.15, TBI at 3-months post-injury**	-0.095	-4.559	**0.004**
	0.15, TBI at 6-months post-injury	-0.067	-3.199	0.362
	0.15, TBI at 12-months post-injury	-0.054	-2.590	0.837
**0.18, Healthy Controls**	**0.18, TBI at 3-months post-injury**	-0.112	-5.389	**<0.0001**

	0.18, TBI at 6-months post-injury	-0.077	-3.681	0.106
	0.18, TBI at 12-months post-injury	-0.064	-3.088	0.449
**0.21, Healthy Controls**	**0.21, TBI at 3-months post-injury**	-0.126	-6.046	**<0.0001**
	**0.21, TBI at 6-months post-injury**	-0.085	-4.062	**0.030**
	0.21, TBI at 12-months post-injury	-0.074	-3.558	0.151
**0.24, Healthy Controls**	**0.24, TBI at 3-months post-injury**	-0.138	-6.631	**<0.0001**
	**0.24, TBI at 6-months post-injury**	-0.092	-4.416	**0.008**
	**0.24, TBI at 12-months post-injury**	-0.084	-4.025	**0.034**
**0.27, Healthy Controls**	**0.27, TBI at 3-months post-injury**	-0.143	-6.848	**<0.0001**
	**0.27, TBI at 6-months post-injury**	-0.099	-4.755	**0.002**
	**0.27, TBI at 12-months post-injury**	-0.093	-4.474	**0.006**

Table S6. Global Amplitude Turbulence in Healthy Controls (mean across session 1 and 2) vs TBI patients (3-,6- and 12-months). Results from the 4 (Group) x 10 (Lambda) ANOVA (top table) and post hoc tests (bottom table). Schaefer parcellation 400 nodes 7 RSNs.

Effect	$F_{(27,440)}$	p-val	Effect size ($\eta^2_{partial}$)
Group x Lambda	3.179	<0.0001	0.163

Lambda, Group	Lambda, Group	Mean Difference	t	pval
**0.01, Healthy Controls**	**0.01, TBI at 3-months post-injury**	0.016	4.635	**0.003**
	**0.01, TBI at 6-months post-injury**	0.026	7.410	**<0.0001**
	**0.01, TBI at 12-months post-injury**	0.018	5.218	**<0.0001**
**0.03, Healthy Controls**	0.03, TBI at 3-months post-injury	0.012	3.605	0.132
	**0.03, TBI at 6-months post-injury**	0.020	5.913	**<0.0001**
	0.03, TBI at 12-months post-injury	0.013	3.637	0.121
0.06, Healthy Controls	0.06, TBI at 3-months post-injury	0.003	0.931	1.000
	0.06, TBI at 6-months post-injury	0.008	2.218	0.974
	0.06, TBI at 12-months post-injury	0.001	0.425	1.000
0.09, Healthy Controls	0.09, TBI at 3-months post-injury	0.003	0.919	1.000
	0.09, TBI at 6-months post-injury	0.005	1.438	1.000
	0.09, TBI at 12-months post-injury	0.001	0.308	1.000
0.12, Healthy Controls	0.12, TBI at 3-months post-injury	0.012	3.574	0.145
	0.12, TBI at 6-months post-injury	0.013	3.747	0.087
	0.12, TBI at 12-months post-injury	0.011	3.191	0.367
**0.15, Healthy Controls**	**0.15, TBI at 3-months post-injury**	0.021	5.994	**<0.0001**
	**0.15, TBI at 6-months post-injury**	0.021	6.035	**<0.0001**
	**0.15, TBI at 12-months post-injury**	0.020	5.945	**<0.0001**
**0.18, Healthy Controls**	**0.18, TBI at 3-months post-injury**	0.024	7.050	**<0.0001**
	**0.18, TBI at 6-months post-injury**	0.024	7.042	**<0.0001**
	**0.18, TBI at 12-months post-injury**	0.025	7.189	**<0.0001**
**0.21, Healthy Controls**	**0.21, TBI at 3-months post-injury**	0.025	7.113	**<0.0001**
	**0.21, TBI at 6-months post-injury**	0.024	7.094	**<0.0001**

	**0.21, TBI at 12-months post-injury**	0.025	7.336	**<0.0001**
**0.24, Healthy Controls**	**0.24, TBI at 3-months post-injury**	0.023	6.571	**<0.0001**
	**0.24, TBI at 6-months post-injury**	0.023	6.558	**<0.0001**
	**0.24, TBI at 12-months post-injury**	0.023	6.809	**<0.0001**
**0.27, Healthy Controls**	**0.27, TBI at 3-months post-injury**	0.020	5.737	**<0.0001**
	**0.27, TBI at 6-months post-injury**	0.020	5.732	**<0.0001**
	**0.27, TBI at 12-months post-injury**	0.021	5.955	**<0.0001**

Table S7. Information Cascade Flow in Healthy Controls (mean across session 1 and 2) vs TBI patients (3-,6- and 12-months). Results from the 4 (Group) x 9 (Lambda) ANOVA (top table) and post hoc tests (bottom table). Schaefer parcellation 400 nodes 7 RSNs.

Effect	$F_{(24,396)}$	p-val	Effect size ($\eta^2_{partial}$)
Group x Lambda	8.358	<0.0001	0.336

Lambda, Group	Lambda, Group	Mean Difference	t	pval
**0.01, Healthy Controls**	**0.01, TBI at 3-months post-injury**	0.096	7.928	**0.000**
	**0.01, TBI at 6-months post-injury**	0.148	12.199	**0.000**
	**0.01, TBI at 12-months post-injury**	0.098	8.096	**0.000**
**0.03, Healthy Controls**	**0.03, TBI at 3-months post-injury**	0.107	8.836	**0.000**
	**0.03, TBI at 6-months post-injury**	0.133	10.973	**0.000**
	**0.03, TBI at 12-months post-injury**	0.105	8.624	**0.000**
**0.06, Healthy Controls**	**0.06, TBI at 3-months post-injury**	0.067	5.502	**<0.001**
	**0.06, TBI at 6-months post-injury**	0.075	6.194	**<0.001**
	**0.06, TBI at 12-months post-injury**	0.064	5.283	**<0.001**
0.09, Healthy Controls	0.09, TBI at 3-months post-injury	0.032	2.605	0.785
	0.09, TBI at 6-months post-injury	0.034	2.821	0.620
	0.09, TBI at 12-months post-injury	0.029	2.393	0.903
0.12, Healthy Controls	0.12, TBI at 3-months post-injury	0.015	1.240	1.000
	0.12, TBI at 6-months post-injury	0.015	1.261	1.000
	0.12, TBI at 12-months post-injury	0.012	1.004	1.000
0.15, Healthy Controls	0.15, TBI at 3-months post-injury	0.009	0.737	1.000
	0.15, TBI at 6-months post-injury	0.009	0.708	1.000
	0.15, TBI at 12-months post-injury	0.006	0.509	1.000
0.18, Healthy Controls	0.18, TBI at 3-months post-injury	0.006	0.530	1.000
	0.18, TBI at 6-months post-injury	0.006	0.482	1.000
	0.18, TBI at 12-months post-injury	0.004	0.316	1.000
0.21, Healthy Controls	0.21, TBI at 3-months post-injury	0.005	0.424	1.000
	0.21, TBI at 6-months post-injury	0.004	0.366	1.000
	0.21, TBI at 12-months post-injury	0.003	0.223	1.000
0.24, Healthy Controls	0.24, TBI at 3-months post-injury	0.004	0.362	1.000
	0.24, TBI at 6-months post-injury	0.004	0.316	1.000
	0.24, TBI at 12-months post-injury	0.002	0.177	1.000

Table S8. Information Cascade in Healthy Controls (mean across session 1 and 2) vs TBI patients (3-,6- and 12-months). Results from the 4 one-way ANOVA (top table) and post hoc tests (bottom table). Schaefer parcellation 400 nodes 7 RSNs.

Effect	$F_{(3,440)}$	p-val	Effect size ($\eta^2_{partial}$)
Group	22.207	<0.0001	0.602

Group 1	Group 2	Mean Difference	t	pval

**Healthy Controls**	**TBI at 3-months post-injury**	0.038	6.059	**<0.0001**
	**TBI at 6-months post-injury**	0.048	7.599	**<0.0001**
	**TBI at 12-months post-injury**	0.036	5.728	**<0.0001**
TBI at 3-months post-injury	TBI at 6-months post-injury	0.010	1.539	0.423
	TBI at 12-months post-injury	-0.002	-0.331	0.987
TBI at 6-months post-injury	TBI at 12-months post-injury	-0.012	-1.871	0.255

Table S9. Information Transfer in Healthy Controls mean across session 1 and 2) vs TBI patients (3-,6- and 12-months). Results from the 4 (Group) x 10 (Lambda) ANOVA (top table) and post hoc tests (bottom table). Schaefer parcellation 400 nodes 7 RSNs.

Effect	$F_{(27,440)}$	p-val	Effect size ($\eta^2_{partial}$)
Group x Lambda	3.305	<0.0001	0.169

**Lambda, Group**	**Lambda, Group**	**Mean Difference**	**t**	pval
0.01, Healthy Controls	0.01, TBI at 3-months post-injury	-0.001	-0.082	1.000
	0.01, TBI at 6-months post-injury	-0.002	-0.122	1.000
	0.01, TBI at 12-months post-injury	-0.002	-0.100	1.000
0.03, Healthy Controls	0.03, TBI at 3-months post-injury	-0.015	-1.001	1.000
	0.03, TBI at 6-months post-injury	-0.021	-1.356	1.000
	0.03, TBI at 12-months post-injury	-0.017	-1.112	1.000
0.06, Healthy Controls	0.06, TBI at 3-months post-injury	-0.051	-3.305	0.288
	0.06, TBI at 6-months post-injury	-0.060	-3.915	0.050
	0.06, TBI at 12-months post-injury	-0.052	-3.342	0.263
**0.09, Healthy Controls**	0.09, TBI at 3-months post-injury	-0.059	-3.801	0.073
	**0.09, TBI at 6-months post-injury**	-0.069	-4.492	**0.006**
	0.09, TBI at 12-months post-injury	-0.058	-3.779	0.078
0.12, Healthy Controls	0.12, TBI at 3-months post-injury	-0.026	-1.691	1.000
	0.12, TBI at 6-months post-injury	-0.037	-2.422	0.919
	0.12, TBI at 12-months post-injury	-0.024	-1.583	1.000
0.15, Healthy Controls	0.15, TBI at 3-months post-injury	0.008	0.520	1.000
	0.15, TBI at 6-months post-injury	-0.003	-0.222	1.000
	0.15, TBI at 12-months post-injury	0.012	0.806	1.000
0.18, Healthy Controls	0.18, TBI at 3-months post-injury	0.026	1.652	1.000
	0.18, TBI at 6-months post-injury	0.015	0.949	1.000
	0.18, TBI at 12-months post-injury	0.033	2.141	0.985
0.21, Healthy Controls	0.21, TBI at 3-months post-injury	0.036	2.306	0.956
	0.21, TBI at 6-months post-injury	0.026	1.659	1.000
	0.21, TBI at 12-months post-injury	0.046	2.994	0.526
0.24, Healthy Controls	0.24, TBI at 3-months post-injury	0.041	2.650	0.798
	0.24, TBI at 6-months post-injury	0.032	2.068	0.991
	0.24, TBI at 12-months post-injury	0.054	3.516	0.169
0.27, Healthy Controls	0.27, TBI at 3-months post-injury	0.043	2.750	0.727
	0.27, TBI at 6-months post-injury	0.035	2.240	0.970

	0.27, TBI at 12-months post-injury	0.058	3.779	0.078

Table S10. Global Amplitude Turbulence in TBI patients over time. Results from the repeated measures ANCOVA with lesion volume as nuisance covariate (top table) and post hoc tests (bottom table).

Effect	$F_{(18,180)}$	p-val	Effect size ($\eta^2_{partial}$)
Time x Lambda	2.073	0.008	0.172

Time, Lambda	Time, Lambda	Mean Difference	t	pval
**TBI at 3-months post injury, 0.01**	**TBI at 6-months post injury, 0.01**	0.010	3.844	**0.018**
	TBI at 12-months post injury, 0.01	2.345×10^{-5}	0.009	1.000
TBI at 3-months post injury, 0.03	TBI at 6-months post injury, 0.03	0.008	2.915	0.312
	TBI at 12-months post injury, 0.03	-0.002	-0.756	1.000
TBI at 3-months post injury, 0.06	TBI at 6-months post injury, 0.06	0.004	1.441	1.000
	TBI at 12-months post injury, 0.06	-0.004	-1.574	1.000
TBI at 3-months post injury, 0.09	TBI at 6-months post injury, 0.09	0.002	0.633	1.000
	TBI at 12-months post injury, 0.09	-0.004	-1.542	1.000
TBI at 3-months post injury, 0.12	TBI at 6-months post injury, 0.12	6.371×10^{-4}	0.243	1.000
	TBI at 12-months post injury, 0.12	-0.003	-1.210	1.000
TBI at 3-months post injury, 0.15	TBI at 6-months post injury, 0.15	1.608×10^{-4}	0.061	1.000
	TBI at 12-months post injury, 0.15	-0.002	-0.716	1.000
TBI at 3-months post injury, 0.18	TBI at 6-months post injury, 0.18	-1.446×10^{-4}	-0.055	1.000
	TBI at 12-months post injury, 0.18	-8.568×10^{-4}	-0.327	1.000
TBI at 3-months post injury, 0.21	TBI at 6-months post injury, 0.21	-3.818×10^{-4}	-0.146	1.000
	TBI at 12-months post injury, 0.21	-2.538×10^{-4}	-0.097	1.000
TBI at 3-months post injury, 0.24	TBI at 6-months post injury, 0.24	-5.478×10^{-4}	-0.209	1.000
	TBI at 12-months post injury, 0.24	7.975×10^{-5}	0.030	1.000
TBI at 3-months post injury, 0.27	TBI at 6-months post injury, 0.27	-6.408×10^{-4}	-0.245	1.000
	TBI at 12-months post injury, 0.27	2.635×10^{-4}	0.101	1.000

Table S11. Information Cascade Flow in TBI patients over time. Results from the repeated measures ANCOVA with lesion volume as nuisance covariate (top table) and post hoc tests (bottom table).

Effect	$F_{(16,160)}$	p-val	Effect size ($\eta^2_{partial}$)
Time x Lambda	1.809	0.034	0.153

Time, Lambda	Time, Lambda	Mean Difference	t	pval
**TBI at 3-months post injury, 0.01**	**TBI at 6-months post injury, 0.01**	0.051	4.733	**<0.0001**
	TBI at 12-months post injury, 0.01	-0.008	-0.719	1.000
TBI at 3-months post injury, 0.03	TBI at 6-months post injury, 0.03	0.027	2.482	1.000
	TBI at 12-months post injury, 0.03	-0.005	-0.447	1.000
TBI at 3-months post injury, 0.06	TBI at 6-months post injury, 0.06	0.009	0.830	1.000

	TBI at 12-months post injury, 0.06	-0.003	-0.311	1.000
TBI at 3-months post injury, 0.09	TBI at 6-months post injury, 0.09	0.003	0.262	1.000
	TBI at 12-months post injury, 0.09	-0.003	-0.256	1.000
TBI at 3-months post injury, 0.12	TBI at 6-months post injury, 0.12	$6.805{\times}10^{-4}$	0.063	1.000
	TBI at 12-months post injury, 0.12	-0.003	-0.250	1.000
TBI at 3-months post injury, 0.15	TBI at 6-months post injury, 0.15	$2.256{\times}10^{-4}$	0.021	1.000
	TBI at 12-months post injury, 0.15	-0.002	-0.219	1.000
TBI at 3-months post injury, 0.18	TBI at 6-months post injury, 0.18	$1.249{\times}10^{-4}$	0.012	1.000
	TBI at 12-months post injury, 0.18	-0.002	-0.195	1.000
TBI at 3-months post injury, 0.21	TBI at 6-months post injury, 0.21	$1.236{\times}10^{-4}$	0.011	1.000
	TBI at 12-months post injury, 0.21	-0.002	-0.173	1.000
TBI at 3-months post injury, 0.24	TBI at 6-months post injury, 0.24	$2.759{\times}10^{-5}$	0.003	1.000
	TBI at 12-months post injury, 0.24	-0.002	-0.158	1.000

Table S12. Information Cascade in TBI patients over time. Results from the repeated measures ANCOVA with lesion volume as nuisance covariate (top table) and post hoc tests (bottom table).

Effect	$F_{(2,20)}$	p-val	Effect size ($\eta^2_{partial}$)
Time	4.205	0.030	0.296

Group 1	Group 2	Mean Difference	t	pval
TBI at 3-months post-injury	TBI at 6-months post-injury	0.010	2.195	0.080
	TBI at 12-months post-injury	-0.003	-0.712	0.485
**TBI at 6-months post-injury**	**TBI at 12-months post-injury**	-0.013	-2.906	**0.026**

Table S13. Information Transfer in TBI patients over time. Non-significant results from the repeated measures ANCOVA with lesion volume as nuisance covariate.

Effect	$F_{(18,180)}$	p-val	Effect size ($\eta^2_{partial}$)
Time x Lambda	0.826	0.668	0.076

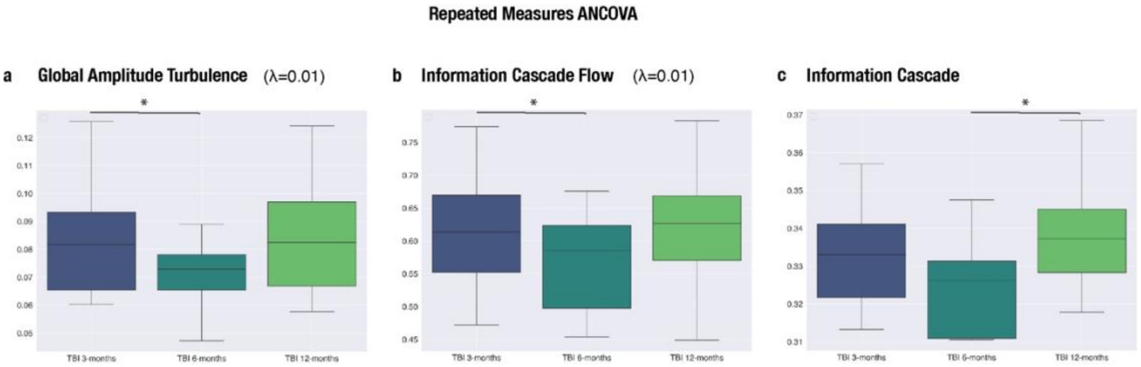

Figure S1. Boxplots depicting the significant results from the repeated measures ANCOVA with lesion volume as nuisance covariate.

Tabla S14. Amplitude turbulence in the 7 resting state networks (RSN) included in the Schaefer 1000 nodes parcellation. Results from the one-way ANOVA (4 groups) for λ=0.01 and λ=0.03 (top table) and post hoc tests for λ=0.03 (bottom table).

Lambda	RSN	$F_{(3,44)}$	p-val	Effect size ($\eta^2_{partial}$)
**0.01**	VIS	2.203	0.101	0.131
**0.01**	SM	2.014	0.126	0.121
**0.01**	DAT	2.211	0.100	0.131
**0.01**	VAT	1.820	0.157	0.110
**0.01**	LIM	1.486	0.231	0.092
**0.01**	CNT	1.931	0.139	0.116
**0.01**	DMN	1.881	0.147	0.114
**0.03**	VIS	2.859	**0.048**	0.163
**0.03**	SM	4.575	**0.007**	0.238
**0.03**	DAT	4.558	**0.007**	0.237
**0.03**	VAT	3.015	**0.040**	0.171
**0.03**	LIM	1.324	0.279	0.083
**0.03**	CNT	4.017	**0.013**	0.215
**0.03**	DMN	3.146	**0.034**	0.177

RSN	Group 1	Group 2	Mean Difference	t	pval
**VIS**	**Healthy Controls**	TBI at 3-months post-injury	0.015	2.244	0.127
		**TBI at 6-months post-injury**	0.019	2.751	**0.041**
		TBI at 12-months post-injury	0.012	1.713	0.329
**SM**	**Healthy Controls**	TBI at 3-months post-injury	0.011	1.983	0.210
		**TBI at 6-months post-injury**	0.021	3.700	**0.003**
		TBI at 12-months post-injury	0.010	1.795	0.289
**DAT**	**Healthy Controls**	TBI at 3-months post-injury	0.014	2.216	0.135
		**TBI at 6-months post-injury**	0.023	3.660	**0.004**
		TBI at 12-months post-injury	0.014	2.190	0.142
**VAT**	**Healthy Controls**	TBI at 3-months post-injury	0.007	1.361	0.530
		**TBI at 6-months post-injury**	0.017	3.002	**0.022**
		TBI at 12-months post-injury	0.008	1.373	0.523
**CNT**	**Healthy Controls**	TBI at 3-months post-injury	0.010	1.875	0.253
		**TBI at 6-months post-injury**	0.019	3.466	**0.006**
		TBI at 12-months post-injury	0.010	1.865	0.258
**DMN**	**Healthy Controls**	TBI at 3-months post-injury	0.010	1.682	0.345
		**TBI at 6-months post-injury**	0.018	3.067	**0.019**
		TBI at 12-months post-injury	0.009	1.554	0.415

Table S15. Node-level amplitude turbulence. Results from the Kolmogorov-Smirnov test.

Difference	Lambda	KSD	pval
HCs-TBI at 3-months post-injury	0.27	0.033	<0.0001
HCs-TBI at 3-months post-injury	0.24	0.0386	<0.0001
HCs-TBI at 3-months post-injury	0.21	0.0506	<0.0001
HCs-TBI at 3-months post-injury	0.18	0.071	<0.0001
HCs-TBI at 3-months post-injury	0.15	0.091	<0.0001
HCs-TBI at 3-months post-injury	0.12	0.124	<0.0001
HCs-TBI at 3-months post-injury	0.09	0.160	<0.0001
HCs-TBI at 3-months post-injury	0.06	0.206	<0.0001
HCs-TBI at 3-months post-injury	0.03	0.242	<0.0001

HCs-TBI at 3-months post-injury	0.01	0.249	<0.0001
HCs-TBI at 6-months post-injury	0.27	0.0317	<0.0001
HCs-TBI at 6-months post-injury	0.24	0.0421	<0.0001
HCs-TBI at 6-months post-injury	0.21	0.0571	<0.0001
HCs-TBI at 6-months post-injury	0.18	0.0802	<0.0001
HCs-TBI at 6-months post-injury	0.15	0.105	<0.0001
HCs-TBI at 6-months post-injury	0.12	0.156	<0.0001
HCs-TBI at 6-months post-injury	0.09	<0.0001	<0.0001
HCs-TBI at 6-months post-injury	0.06	0.303	0
**HCs-TBI at 6-months post-injury**	**0.03**	**0.420**	0
**HCs-TBI at 6-months post-injury**	**0.01**	**0.494**	0
HCs-TBI at 12-months post-injury	0.27	0.0345	<0.0001
HCs-TBI at 12-months post-injury	0.24	0.0389	<0.0001
HCs-TBI at 12-months post-injury	0.21	0.0462	<0.0001
HCs-TBI at 12-months post-injury	0.18	0.0628	<0.0001
HCs-TBI at 12-months post-injury	0.15	0.074	<0.0001
HCs-TBI at 12-months post-injury	0.12	0.092	<0.0001
HCs-TBI at 12-months post-injury	0.09	0.112	<0.0001
HCs-TBI at 12-months post-injury	0.06	0.141	<0.0001
HCs-TBI at 12-months post-injury	0.03	0.209	<0.0001
HCs-TBI at 12-months post-injury	0.01	0.294	0

Table S16. Correlation between turbulent-like dynamics measures and cognitive performance in TBI patients at 3-months post-injury.

	**Stroop C total**		**Stroop CW total**		**TMTA**		**TMTB**		**Dig Total**		**VSAT total**	
**Turbulent measure**	**r**	**p**	**r**	**p**	**r**	**p**	**r**	**p**	**r**	**p**	**r**	**p**
Turbulence Global λ=0.06	0.372	0.260	0.623	0.054	0.254	0.451	0.198	0.583	0.679	0.022	0.275	0.412
Turbulence Global λ=0.03	0.416	0.203	0.548	0.101	0.235	0.486	0.267	0.456	0.711	0.014	0.163	0.633
Turbulence Global λ=0.01	0.442	0.173	0.443	0.200	0.181	0.595	0.331	0.351	0.685	0.020	0.093	0.785
Info Casc Flow λ=0.01	0.584	0.059	0.346	0.327	0.159	0.641	0.206	0.569	0.754	**0.007***	-0.047	0.891
Info Transfer λ=0.27	-0.059	0.864	0.120	0.741	0.360	0.276	0.324	0.360	-0.023	0.945	0.196	0.563
Info Transfer λ=0.24	-0.034	0.921	0.128	0.725	0.433	0.184	0.391	0.264	-0.038	0.911	0.217	0.521
Info Transfer λ=0.21	-0.047	0.891	0.101	0.780	0.424	0.194	0.372	0.290	-0.004	0.991	0.196	0.563
Info Transfer λ=0.18	-0.059	0.863	0.062	0.865	0.415	0.204	0.343	0.332	0.014	0.967	0.177	0.602
Info Transfer λ=0.15	-0.094	0.784	0.011	0.976	0.415	0.204	0.276	0.441	-0.007	0.985	0.158	0.642

The variables r and p denote the Pearson correlation coefficient and associated p-value, respectively. The asterisk (*) denotes that the correlation survived FDR correction for multiple comparisons across tasks (p-adj < 0.05). C: Color, CW: Color-Word, TMTA: Trail Making Test A, TMTB: Trail Making Test B, Dig: Digit Span Forward and Backward, VSAT: Visual Search and Attention Test.

Table S17. Correlation between turbulent-like dynamics measures by resting-state network (RSN) and cognitive performance in TBI patients at 3-months post-injury.

	**Stroop C total**		**Stroop CW total**		**TMTA**		**TMTB**		**Dig Total**		**VSAT total**	
**Turbulent measure by RSN**	**r**	**p**	**r**	**p**	**r**	**p**	**r**	**p**	**r**	**p**	**r**	**p**
Turbulence VIS λ=0.03	0.526	0.096	0.728	0.017	0.153	0.653	0.157	0.665	0.689	0.019	0.260	0.440
Turbulence SM λ=0.03	0.375	0.413	0.392	0.263	0.306	0.360	0.395	0.259	0.679	0.022	0.147	0.665
Turbulence DAT λ=0.03	0.256	0.448	0.545	0.103	0.273	0.417	0.267	0.456	0.614	0.044	0.211	0.534
Turbulence VAT λ=0.03	0.416	0.203	0.436	0.208	0.275	0.413	0.346	0.328	0.721	0.012	0.111	0.746
Turbulence LIM λ=0.03	0.521	0.100	0.412	0.236	0.136	0.691	0.114	0.754	0.675	0.023	0.046	0.893
Turbulence CON λ=0.03	0.374	0.257	0.496	0.145	0.218	0.520	0.272	0.447	0.633	0.036	0.178	0.600
Turbulence DMN λ=0.03	0.495	0.121	0.491	0.150	0.163	0.632	0.163	0.653	0.744	**0.009***	0.081	0.813

The variables r and p denote the Pearson correlation coefficient and associated p-value, respectively. The asterisk (*) denotes that the correlation survived FDR correction for multiple comparisons across tasks (p-adj < 0.05). C: Color, CW: Color-Word, TMTA: Trail Making Test A, TMTB: Trail Making Test B, Dig: Digit Span Forward and Backward, VSAT: Visual Search and Attention Test.

Table S18. Global coupling parameter after the simulated attack approach. SC: structural connectivity (see Material and Methods).

	Intact SC	SC with lesion mask 1.5SD (binary attack)	SC with lesion mask 1.5SD (weighted attack)	SC with lesion mask 2SD (binary attack)	SC with lesion mask 2SD (weighted attack)
TBI 3-months	0.33	0.32	0.31	0.32	0.31
TBI 6-months	**0.31**	**0.30**	**0.29**	**0.30**	**0.29**
TBI 12-months	0.32	0.32	0.31	0.32	0.31

Table S19. Anatomical regions lesions in the structural connectivity matrix thresholded at 1.5SD. The node label is indicated along with the corresponding anatomical region (see Material and Methods).

Node to attack Schaefer 1000 nodes label	Anatomical region	Frequency (TBI Patients with lesion)
143	7Networks_LH_SomMot_62\	1
155	7Networks_LH_SomMot_74\	1
161	7Networks_LH_SomMot_80\	1
189	7Networks_LH_DorsAttn_Post_17\	1
245	7Networks_LH_SalVentAttn_FrOperIns_1\	1
249	7Networks_LH_SalVentAttn_FrOperIns_5\	1
259	7Networks_LH_SalVentAttn_FrOperIns_15\	1
274	7Networks_LH_SalVentAttn_Med_5\	1
284	7Networks_LH_SalVentAttn_Med_15\	1
292	7Networks_LH_Limbic_OFC_4\	1
367	7Networks_LH_Cont_Cing_3\	1
368	7Networks_LH_Cont_Cing_4\	1
370	7Networks_LH_Cont_Cing_6\	1
416	7Networks_LH_Default_PFC_1\	2
417	7Networks_LH_Default_PFC_2\	1
471	7Networks_LH_Default_pCunPCC_6\	1
497	7Networks_LH_Default_pCunPCC_32\	1
533	7Networks_RH_Vis_33\	1
536	7Networks_RH_Vis_36\	1
555	7Networks_RH_Vis_55\	1
586	7Networks_RH_SomMot_5\	1
591	7Networks_RH_SomMot_10\	1
598	7Networks_RH_SomMot_17\	1
612	7Networks_RH_SomMot_31\	1
618	7Networks_RH_SomMot_37\	1
632	7Networks_RH_SomMot_51\	1
671	7Networks_RH_SomMot_90\	1

679	7Networks_RH_SomMot_98\	1
717	7Networks_RH_DorsAttn_Post_33\	1
722	7Networks_RH_DorsAttn_Post_38\	1
753	7Networks_RH_SalVentAttn_TempOccPar_8\	1
775	7Networks_RH_SalVentAttn_FrOperIns_10\	1
805	7Networks_RH_SalVentAttn_Med_14\	1
842	7Networks_RH_Limbic_TempPole_17\	1
843	7Networks_RH_Cont_Par_1\	1
865	7Networks_RH_Cont_PFCv_2\	2
913	7Networks_RH_Default_Par_1\	1
919	7Networks_RH_Default_Par_7\	1
949	7Networks_RH_Default_PFCv_3\	1
961	7Networks_RH_Default_PFCdPFCm_5\	1
999	7Networks_RH_Cont_pCun_2\	1

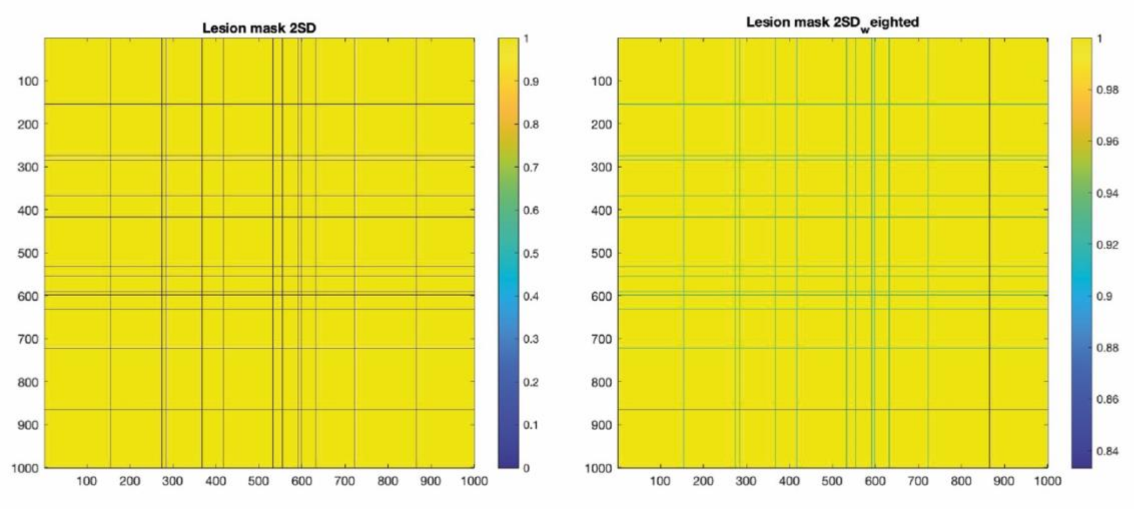

Figure S2. Heatmaps for the lesion mask thresholded at 2SD with the binary (left) and weighted (right) approach for the simulated attack (see Material and Methods).

Table S20. Susceptibility in HCs (session 1 and 2) vs TBI (3-,6- and 12-months post-injury). Results from the two-sided Wilcoxon rank sum test. Lesion mask 1.5 SD (see Material and Methods).

Group comparison	Z	pval	padj
HCs s1 vs TBI 3mo	6.29	<0.0001	<0.0001
HCs s1 vs TBI 6mo	10.77	<0.0001	<0.0001
HCs s1 vs TBI 12mo	10.25	<0.0001	<0.0001
HCs s1 vs TBI 3mo lesion 1.5 SD Binary	10.25	<0.0001	<0.0001
HCs s1 vs TBI 6mo lesion 1.5 SD Binary	11.89	<0.0001	<0.0001
HCs s1 vs TBI 12mo lesion 1.5 SD Binary	11.58	<0.0001	<0.0001
HCs s1 vs TBI 3mo lesion 1.5 SD Weighted	7.09	<0.0001	<0.0001
HCs s1 vs TBI 6mo lesion 1.5 SD Weighted	11.09	<0.0001	<0.0001
HCs s1 vs TBI 12mo lesion 1.5 SD Weighted	10	<0.0001	<0.0001
HCs s2 vs TBI 3mo	2.80	0.0051	0.0051
HCs s2 vs TBI 6mo	9.04	<0.0001	<0.0001
HCs s2 vs TBI 12mo	8.03	<0.0001	<0.0001
HCs s2 vs TBI 3mo lesion 1.5 SD Binary	8.21	<0.0001	<0.0001
HCs s2 vs TBI 6mo lesion 1.5 SD Binary	11.44	<0.0001	<0.0001
HCs s2 vs TBI 12mo lesion 1.5 SD Binary	10.71	<0.0001	<0.0001
HCs s2 vs TBI 3mo lesion 1.5 SD Weighted	3.83	<0.0001	<0.0001

HCs s2 vs TBI 6mo lesion 1.5 SD Weighted	9.68	<0.0001	<0.0001
HCs s2 vs TBI 12mo lesion 1.5 SD Weighted	7.66	<0.0001	<0.0001

Table S21. Information encoding capability in HCs (session 1 and 2) vs TBI (3-,6- and 12-months post-injury). Results from the two-sided Wilcoxon rank sum test. Lesion mask 1.5 SD (see Material and Methods).

Group comparison	Z	pval	padj
HCs s1 vs TBI 3mo	8.35	<0.0001	<0.0001
HCs s1 vs TBI 6mo	12.02	<0.0001	<0.0001
HCs s1 vs TBI 12mo	11.82	<0.0001	<0.0001
HCs s1 vs TBI 3mo lesion 1.5 SD Binary	10.72	<0.0001	<0.0001
HCs s1 vs TBI 6mo lesion 1.5 SD Binary	12.18	<0.0001	<0.0001
HCs s1 vs TBI 12mo lesion 1.5 SD Binary	12.07	<0.0001	<0.0001
HCs s1 vs TBI 3mo lesion 1.5 SD Weighted	9.25	<0.0001	<0.0001
HCs s1 vs TBI 6mo lesion 1.5 SD Weighted	12.10	<0.0001	<0.0001
HCs s1 vs TBI 12mo lesion 1.5 SD Weighted	11.71	<0.0001	<0.0001
HCs s2 vs TBI 3mo	4.59	<0.0001	<0.0001
HCs s2 vs TBI 6mo	11.36	<0.0001	<0.0001
HCs s2 vs TBI 12mo	10.80	<0.0001	<0.0001
HCs s2 vs TBI 3mo lesion 1.5 SD Binary	8.64	<0.0001	<0.0001
HCs s2 vs TBI 6mo lesion 1.5 SD Binary	12.04	<0.0001	<0.0001
HCs s2 vs TBI 12mo lesion 1.5 SD Binary	11.60	<0.0001	<0.0001
HCs s2 vs TBI 3mo lesion 1.5 SD Weighted	5.86	<0.0001	<0.0001
HCs s2 vs TBI 6mo lesion 1.5 SD Weighted	11.73	<0.0001	<0.0001
HCs s2 vs TBI 12mo lesion 1.5 SD Weighted	10.49	<0.0001	<0.0001

Table S22. Susceptibility in HCs (session 1 and 2) vs TBI (3-,6- and 12-months post-injury). Results from the two-sided Wilcoxon rank sum test. Lesion mask 2 SD (see Material and Methods).

Group comparison	Z	pval	padj
HCs s1 vs TBI 3mo	6.29	<0.0001	<0.0001
HCs s1 vs TBI 6mo	10.77	<0.0001	<0.0001
HCs s1 vs TBI 12mo	10.25	<0.0001	<0.0001
HCs s1 vs TBI 3mo lesion 2 SD Binary	8.54	<0.0001	<0.0001
HCs s1 vs TBI 6mo lesion 2 SD Binary	11.46	<0.0001	<0.0001
HCs s1 vs TBI 12mo lesion 2 SD Binary	10.80	<0.0001	<0.0001
HCs s1 vs TBI 3mo lesion 2 SD Weighted	6.93	<0.0001	<0.0001
HCs s1 vs TBI 6mo lesion 2 SD Weighted	11.06	<0.0001	<0.0001
HCs s1 vs TBI 12mo lesion 2 SD Weighted	9.90	<0.0001	<0.0001
HCs s2 vs TBI 3mo	2.80	0.0051	0.0051
HCs s2 vs TBI 6mo	9.04	<0.0001	<0.0001
HCs s2 vs TBI 12mo	8.03	<0.0001	<0.0001
HCs s2 vs TBI 3mo lesion 2 SD Binary	5.76	<0.0001	<0.0001
HCs s2 vs TBI 6mo lesion 2 SD Binary	10.49	<0.0001	<0.0001
HCs s2 vs TBI 12mo lesion 2 SD Binary	9.17	<0.0001	<0.0001
HCs s2 vs TBI 3mo lesion 2 SD Weighted	3.63	0.0002	<0.0001
HCs s2 vs TBI 6mo lesion 2 SD Weighted	9.61	<0.0001	<0.0001
HCs s2 vs TBI 12mo lesion 2 SD Weighted	7.49	<0.0001	<0.0001

Table S23. Information encoding capability in HCs (session 1 and 2) vs TBI (3-,6- and 12-months post-injury). Results from the two-sided Wilcoxon rank sum test. Lesion mask 2 SD (see Material and Methods).

Group comparison	Z	pval	padj
HCs s1 vs TBI 3mo	8.35	<0.0001	<0.0001
HCs s1 vs TBI 6mo	12.02	<0.0001	<0.0001
HCs s1 vs TBI 12mo	11.82	<0.0001	<0.0001
HCs s1 vs TBI 3mo lesion 2 SD Binary	9.66	<0.0001	<0.0001
HCs s1 vs TBI 6mo lesion 2 SD Binary	12.11	<0.0001	<0.0001
HCs s1 vs TBI 12mo lesion 2 SD Binary	11.83	<0.0001	<0.0001
HCs s1 vs TBI 3mo lesion 2 SD Weighted	9.09	<0.0001	<0.0001
HCs s1 vs TBI 6mo lesion 2 SD Weighted	12.09	<0.0001	<0.0001
HCs s1 vs TBI 12mo lesion 2 SD Weighted	11.70	<0.0001	<0.0001
HCs s2 vs TBI 3mo	4.59	<0.0001	<0.0001

HCs s2 vs TBI 6mo	11.36	<0.0001	<0.0001
HCs s2 vs TBI 12mo	10.80	<0.0001	<0.0001
HCs s2 vs TBI 3mo lesion 2 SD Binary	6.76	<0.0001	<0.0001
HCs s2 vs TBI 6mo lesion 2 SD Binary	11.79	<0.0001	<0.0001
HCs s2 vs TBI 12mo lesion 2 SD Binary	10.87	<0.0001	<0.0001
HCs s2 vs TBI 3mo lesion 2 SD Weighted	5.67	<0.0001	<0.0001
HCs s2 vs TBI 6mo lesion 2 SD Weighted	11.69	<0.0001	<0.0001
HCs s2 vs TBI 12mo lesion 2 SD Weighted	10.41	<0.0001	<0.0001

References

Behzadi, Y., Restom, K., Liau, J. and Liu, T.T., 2007. A component-based noise correction method 600(CompCor) for BOLD and perfusion-based fMRI. Neuroimage, 37(1), pp.90-101.

Nieto-Castanon, A., 2020. Handbook of functional connectivity Magnetic Resonance Imaging methods in CONN. Hilbert Press.

Power, J.D., 2017. A simple but useful way to assess fMRI scan qualities. Neuroimage, 154, pp.150-676158.

Whitfield-Gabrieli, S., Nieto-Castanon, A., & Ghosh, S. (2011). Artifact detection tools (ART). 694Cambridge, MA. Release Version 2011.
